# Evaluation of Nurses' Intent to Leave: Italian Translation, Cultural Adaptation, and Initial Psychometric Assessment of the Anticipated Turnover Scale

**DOI:** 10.1002/nop2.70848

**Published:** 2026-10-01

**Authors:** Sara Cignola, Stefano Bembich, Gianfranco Sanson

**Affiliations:** ^1^ Department of Medicine, Surgery and Health Sciences University of Trieste Trieste Italy; ^2^ Unit of Neonatology University Hospital of Udine Udine Italy

**Keywords:** intention to leave, nursing, turnover, validation study

## Abstract

**Aim:**

The aim of this study was to translate, culturally adapt, and preliminarily validate the Italian version of the Anticipated Turnover Scale (ATS).

**Design:**

Cross‐sectional validation study.

**Methods:**

The Italian version of the ATS instrument was evaluated in a non‐probabilistic sample of 168 nurses working at a Northeastern Italian Public University Hospital. Content validity was assessed by expert evaluation and statistical significance analysed using a binomial test. Construct validity was explored through Principal Axis Factoring analysis and internal consistency was measured using Cronbach's alpha.

**Results:**

In a sample of 168 Italian nurses, the Italian 12‐item (complete) version of the ATS showed an Item‐Level Content Validity Index of 0.68, an internal structure with three main factors explaining 57.82% of the variance, and an internal consistency of 0.828. A reduced 10‐item version showed an Item‐Level Content Validity Index of 0.78, an internal structure with two main factors explaining 59.16% of the variance, and internal consistency increased to 0.901.

**Conclusions:**

The Italian version of the ATS was preliminarily evaluated for its validity and reliability. In the current form, it is not ready for practical application yet and further studies should deepen its psychometric properties, as well as perform a confirmatory factor analysis on the factor structures reported here. They should also examine its temporal stability and establish a cut‐off point to estimate actual turnover.

**Implications for Nursing Practice:**

After implementing the questionnaire validation process, as reported above, ATS may help nursing managers to assess intention to leave and plan targeted and personalized interventions for improving retention, engagement, and well‐being among nursing professionals.

**No Patient or Public Contribution:**

No patients, service users, caregivers, or members of the public were involved in this study, as it focused on the psychometric validation of a scale among registered nurses.

## 1 Introduction

The global shortage of nurses is a critical health concern and a major challenge for healthcare systems worldwide (Maleki et al. 2023). This issue is further exacerbated by factors such as an aging population, a greater number of people with chronic illnesses and consequently increased healthcare needs, as well as unfavourable working conditions (Koehler and Olds 2022; Liu et al. 2023; Sul and Lucas 2020). According to estimates by the World Health Organization and the International Council of Nurses, by 2030, there will be a global shortfall of approximately 9 million nurses (Sul and Lucas 2020).

Turnover can be defined as the replacement of staff within an organization, taking into account the balance between outgoing personnel and the arrival of new professionals. It involves both voluntary and involuntary movements, as well as internal and external shifts, including transfers between hospitals or within different departments of the same hospital (Bae 2022). The current literature highlights a substantial increase in nurses' turnover rates (Al Muharraq et al. 2022). Nurse turnover has relevant organizational, clinical, and economic consequences, as the loss of experienced professionals may reduce intellectual capital, productivity, employee performance and efficiency, also affecting staff safety, patient satisfaction, quality of care, and hospital length of stay (Bae 2022; Treglown et al. 2018; Smama'h et al. 2023). Furthermore, healthcare organizations should allocate substantial resources to recruit, hire, and train new nurses to replace those who leave (Bae 2022; Smama'h et al. 2023). Although turnover may create opportunities for motivated new professionals to join the team and contribute to innovation in the medium term, the departure of nurses from a work environment may have immediate negative consequences for those who remain. Specifically, it may increase their workload, reduce job satisfaction, and weaken team relationships and cohesion (Sul and Lucas 2020).

Nurses' intention to leave (ITL) refers to the cognitive and emotional process of considering, planning, and deciding to leave their job, representing a critical stage preceding actual departure (Tadesse et al. 2023). Several factors influence ITL among nurses, including job dissatisfaction, burnout, stress, work‐life conflict, low salary, and lack of professional and emotional engagement (Maleki et al. 2023). Additional determinants include patient safety issues (Sul and Lucas 2020), leadership styles (Niinihuhta and Häggman‐Laitila 2022; Smama'h et al. 2023) and toxic leadership, which can increase organizational mistrust (Türkmen Keskin and Özduyan Kiliç 2024). In recent years, the COVID‐19 pandemic has further exacerbated nurses' intention to leave, with one‐third of nurses considering leaving the profession due to burnout and psychological distress (Ulupınar and Erden 2024). While admitting all the limitations of this categorization, generational differences in turnover have also been reported, with Millennials being more likely to change jobs than Baby Boomers and Generation X (Dols et al. 2019; Koehler and Olds 2022). Across generations, workload and staffing shortages are among the main reasons for turnover, whereas leadership support and salary play a crucial role in retention (Koehler and Olds 2022).

Research about nurse turnover should begin with the analysis of ITL, as turnover intention is important for identifying and anticipating actual turnover and is strongly associated with actually leaving the job (Sul and Lucas 2020). Moreover, providing nursing managers with tools to assess nurses' turnover intentions is essential for effectively managing human resources and maintaining stability in healthcare settings (Sul and Lucas 2020). However, no reliable and valid Italian‐language measurement tool is currently available. Among the available instruments, the Anticipated Turnover Scale (ATS), widely used for its simplicity, reliability, and usability, is a valid tool for assessing nurses' intentions to leave their current work position (Sul and Lucas 2020). Therefore, this study aimed to translate, culturally adapt, and preliminarily validate the ATS for the Italian nursing population, supporting its future use by nurse managers and researchers.

## 2 Methods

### 2.1 Study Design

A translation with cross‐cultural adaptation of the ATS was undertaken, after verifying that no translation of the ATS into Italian had previously been completed or was in progress. Subsequently, a cross‐sectional study was conducted to provide an initial psychometric properties evaluation of the tool for the Italian context.

### 2.2 The Anticipated Turnover Scale (ATS)

The ATS, developed by Hinshaw and Atwood in 1983, assesses nurses' intentions to voluntarily leave their positions, whether through internal or external turnover. The ATS consists of 12 items rated on a Likert scale ranging from 1 (strongly disagree) to 7 (strongly agree), with total scores ranging from 12 to 84. A higher score indicates a greater intention to leave one's position, while a lower score indicates a lesser intention. The scale includes an equal number of positively and negatively worded items to minimize response bias, and its completion takes approximately 5 min (Sul and Lucas 2020).

Regarding psychometric properties, the original study by Hinshaw and Atwood reported a Cronbach's alpha of 0.84 for internal consistency, and factor analysis revealed two main factors explaining 54.9% of the variance (Sul and Lucas 2020). In a study of newly graduated nurses, Cheng et al. utilized the ATS with a 5‐point Likert scale, ranging from 1 (strongly disagree) to 5 (strongly agree), achieving a Cronbach's alpha coefficient of 0.90 and a factor analysis explaining 70.24% of the variance (Cheng et al. 2014).

### 2.3 Translation and Cultural Adaptation

Official authorization to translate, culturally adapt, and reproduce the ATS was obtained from the authors, Jan R. Atwood and Ada Sue Hinshaw, including permission to use the instrument with a 5‐point Likert scale instead of the original 7‐point scale. In fact, following this consultation, the 5‐point format was selected to improve simplicity and usability for respondents, consistent with its effective use in previous studies.

Translation and cultural adaptation of the ATS followed a four‐steps process. First, according to literature recommendation (Beaton et al. 2000; Gélinas et al. 2008; Ljungberg et al. 2015; Sousa and Rojjanasrirat 2011), this process involved four translators with the following profiles: (1) a certified translator with no healthcare background; (2) a non‐healthcare translator with bicultural (Italian‐American) experience; (3) a healthcare translator (nurse); and (4) a healthcare translator (nurse) with bicultural (Italian‐English) experience. All were instructed to translate the instrument and its instructions, noting any issues.

Second, the translated documents were reviewed by a bilingual committee composed—in addition to the research team—of experts in the field (Ljungberg et al. 2015): a Director of Health Professions, a Departmental Nursing Manager, a Nursing Coordinator, a Labor Sociologist, a Psychologist, and an Associate Professor of Nursing. The committee applied a decentralized approach to ensure meaning was preserved in both languages (Ljungberg et al. 2015; Sousa and Rojjanasrirat 2011). They assessed the translated instrument for clarity and completeness, making necessary adjustments after appropriate discussions.

Third, the instrument was back‐translated into English by two independent native English‐speaking translators—one with and one without healthcare background—who were blinded to the original version and its translation (Sousa and Rojjanasrirat 2011).

Finally, the two resulting English versions were matched, reviewed, and discussed by the research team, which proposed the final version, later confirmed by the original author (Zeffiro et al. 2021).

### 2.4 Italian Preliminary Validation of the ATS

The process of preliminary validation was carried out by submitting the so‐built Italian version of the ATS to a hospital nurses' population working at a Northeastern Italian Public University Hospital, which demographic and professional characteristics are reported in a (Table S1). Participants were adequately informed about the study's purpose and provided their informed consent to participate. Data were managed anonymously through the EuSurvey platform, ensuring the security and appropriate use of the information obtained. The study was approved by the Board of Directors of the selected hospital.

All hospital nurses were invited to participate in the study. A self‐administered questionnaire was distributed via email to the registered nurses who provided informed consent and were deemed eligible for inclusion. Freelance nurses and those with managerial positions were excluded from the study. Demographic and occupational data were collected as well. To minimize response bias, the ATS was administered first, followed by questions regarding socio‐demographic and occupational characteristics. Data collection was conducted between October 31 and November 20, 2023. The questionnaire was considered valid only if a response was provided for each item. Among the 300 distributed questionnaires, 172 were returned and 168 were considered valid for inclusion.

### 2.5 Data Analysis

Several aspects of the instrument's psychometric properties were considered. A panel of experts determined: (1) ATS apparent validity in measuring the intended construct (face validity); and (2) if the items included in the instrument adequately represented the domain of the construct (content validity). Face validity was evaluated based on the initial impression regarding whether the instrument appeared to measure what it was intended to measure. To explore content validity, the panel of experts evaluated all items of the translated instrument on a scale from 1 to 4, based on its relevance to the construct. Subsequently, the assigned scores were converted into binary values, grouping responses as ‘relevant’ (scores 3–4) or ‘not relevant’ (scores 1–2). The binomial distribution of responses obtained was then analysed by the binomial test to evaluate the content validity of each item (Dalawi et al. 2023; Gélinas et al. 2008; Ljungberg et al. 2015). The statistical significance was set at *p* < 0.05. The Item‐Level Content Validity Index (I‐CVI) and the Scale‐Level Content Validity Index (S‐CVI) were also calculated.

The internal structure of the instrument was explored through factor analysis (Gélinas et al. 2008). The Principal Axis Factoring (PAF) technique with Promax rotation (kappa value = 4) was used, including all the 12 items in the model. The PAF was chosen to identify a possible set of underlying variables with a causal role in responding to the ATS. Only factors with an eigenvalue > 1 and factor loadings > 0.30 were taken into account for interpreting the extracted factor structure and its contents.

Reliability of the instrument was assessed evaluating its internal consistency, as measured by Cronbach's Alpha coefficient. A value > 0.80 was considered acceptable to express a good internal consistency. All statistical analyses were performed using the software SPSS version 22.0 for Windows (Armonk, NY, IBM Corp.).

## 3 Results

### 3.1 Face Validity

The panel of experts assessed the face validity of the instrument, conducting a subjective review to evaluate its ability to capture nurses' turnover intentions. Criteria considered by experts were: clarity, comprehensibility/readability, appropriateness, and relevance. The broadly expressed opinion suggests that the tool is able to capture the phenomenon of nursing turnover effectively with no modifications. The panel thus confirmed its face validity and highlighted its potential for application in both research and practice.

### 3.2 Content Validity

Items 2 and 12 showed higher agreement among evaluators, being rated by everyone as relevant (scores 3–4) and yielding a *p*‐value = 0.031 in the binomial test. None of the other items reached statistical significance among experts, being items 7 and 9 deemed not relevant by five out of six evaluators. A S‐CVI value of 0.68 was found (average agreement); after exclusion of items 7 and 9, it increased to a value of 0.78. Content validity results are reported in Table 1, which also includes the I‐CVIs.

**TABLE 1:** Experts' panel ratings of content validity.

Item	Score 1–2 (*n* = 6)	Score 3–4 (*n* = 6)	Proportion score 1–2	Proportion score 3–4 (I‐CVI)	*p*‐value
1	1	5	0.17	0.83	0.22
2	0	6	0.00	1.00	0.031
3	2	4	0.33	0.67	0.69
4	2	4	0.33	0.67	0.69
5	1	5	0.17	0.83	0.22
6	1	5	0.17	0.83	0.22
7	5	1	0.83	0.17	0.22
8	3	3	0.50	0.50	1.00
9	5	1	0.83	0.17	0.22
10	2	4	0.33	0.67	0.69
11	1	5	0.17	0.83	0.22
12	0	6	0.00	1.00	0.031

Abbreviation: I‐CVI, Item‐Level Content Validity Index.

### 3.3 Internal Structure

The collected data were considered adequate to be explored by factor analysis, as very good outcomes were observed in Kaiser‐Meyer‐Olkin value (0.891) and Bartelett's test of sphericity (*p* < 0.001) (Gray et al. 2017).

The PAF identified three main factors with eigenvalues greater than 1. However, after Promax rotation, the third factor exhibited an eigenvalue < 1 (0.83) and the extracted factorial solution accounted for 57.82% of the variance (Table 2). In the first extracted factor (eigenvalue = 5.23, explained variance = 44.59%), all items loaded positively with values greater than 0.30, except for item 4 (positive loading, with a value < 0.30), item 7 (negative loading, with a value > 0.30), item 9 (negative loading, with a value < 0.30), and item 11 (negative loading, with a value < 0.30). In the second extracted factor (eigenvalue = 2.96; explained variance = 8.20%), item 11 fully loaded with a value of 1.0 and items 4–5 loaded with values slightly > 0.30, all with a positive sign. Finally, in the third factor (eigenvalue = 0.83; explained variance = 5.03%), only item 9 loaded positively with a value > 0.30 (Table 3).

**TABLE 2:** Factor analysis result (Principal Axis Factoring) of the Italian 12‐item version of ATS.

Item	Initial eigenvalues			Rotation sums of squared loadings		
	Total	Variance %	Cumulative %	Total	Variance %	Cumulative %
1	5.71	47.61	47.61	5.23	44.59	44.59
2	1.29	10.71	58.32	2.96	8.20	52.80
3	1.01	8.44	66.76	0.83	5.03	57.82
4	0.84	6.97	73.73			
5	0.76	6.33	80.06			
6	0.59	4.93	84.98			
7	0.45	3.75	88.73			
8	0.37	3.09	91.82			
9	0.29	2.40	94.22			
10	0.28	2.30	96.51			
11	0.24	1.96	98.47			
12	0.18	1.53	100.00			

**TABLE 3:** Item loadings on the three main factors extracted from the Italian 12‐item version of ATS, after Promax rotation (kappa value = 4).

Item	Factor		
	1	2	3
1	0.895	−0.079	0.034
2	0.836	−0.080	−0.111
3	0.509	−0.009	−0.073
4	0.217	0.379	0.244
5	0.458	0.364	−0.132
6	0.904	−0.026	−0.139
7	−0.422	0.010	0.112
8	0.749	0.040	0.130
9	−0.103	−0.057	0.759
10	0.606	0.185	0.215
11	−0.123	1.000	−0.114
12	0.864	0.008	0.018

Basing on such results and taking into account the content validity feedback, items 7 and 9, deemed irrelevant by 5 out of 6 experts, were excluded from a second factor analysis. This further PAF included only 10 items and revealed two main factors with eigenvalues > 1, explaining 59.16% of the variance (Table 4). However, after Promax rotation, the second extracted factor exhibited an eigenvalue < 1 (0.76). The first factor (Eigenvalue = 5.04, explained variance = 51.57%) included all items with positive saturations > 0.30, with the exception of item 4 (positive loading, with a value < 0.30) and item 11 (negative loading, with a value < 0.30). The second factor (eigenvalue = 2.74, explained variance = 7.59%) included only item 5 and item 11, both with a positive saturation > 0.30 (Table 5).

**TABLE 4:** Factor analysis result (Principal Axis Factoring) of the Italian 10‐item version of ATS.

Item	Initial eigenvalues			Rotation sums of squared loadings		
	Total	Variance %	Cumulative %	Total	Variance %	Cumulative %
1	5.52	55.24	55.24	5.16	51.57	51.57
2	1.03	10.29	65.53	0.76	7.59	59.16
3	0.80	7.96	73.49			
4	0.71	7.10	80.59			
5	0.54	5.42	86.01			
6	0.38	3.75	89.76			
7	0.31	3.14	92.90			
8	0.28	2.78	95.68			
9	0.24	2.41	98.09			
10	0.19	1.91	100			

**TABLE 5:** Item loadings on the two main factors extracted from the Italian 10‐item version of ATS, after Promax rotation (kappa value = 4).

Item	Factor	
	1	2
1	0.911	−0.095
2	0.779	−0.017
3	0.489	0.020
4	0.292	0.294
5	0.417	0.421
6	0.846	0.039
8	0.785	0.005
10	0.655	0.125
11	−0.107	0.975
12	0.874	0.003

### 3.4 Reliability

Internal consistency was assessed using Cronbach's alpha. For the full 12‐item ATS scale, Cronbach's alpha was 0.828. However, excluding again items 7 and 9, Cronbach's alpha increased to 0.901. Table 6 and Table 7 report Cronbach's alpha coefficient values for, respectively, the 12‐ and 10‐item factor analysis performed.

**TABLE 6:** Cronbach's alpha values for each item of the Italian 12‐item version of ATS.

Item	Corrected item‐total correlation	Cronbach's alpha excluding the item
1	0.752	0.793
2	0.672	0.799
3	0.461	0.817
4	0.496	0.815
5	0.631	0.804
6	0.770	0.789
7	−0.374	0.877
8	0.726	0.799
9	−0.095	0.852
10	0.694	0.802
11	0.437	0.820
12	0.794	0.788

**TABLE 7:** Cronbach's alpha values for each item of the Italian 10‐item version of ATS.

Item	Corrected item‐total correlation	Cronbach's alpha excluding the item
1	0.769	0.884
2	0.707	0.888
3	0.475	0.903
4	0.476	0.904
5	0.667	0.891
6	0.813	0.880
8	0.724	0.888
10	0.691	0.890
11	0.481	0.904
12	0.812	0.881

Internal consistency was also calculated for PAF extracted factors, where appropriate (e.g., the first extracted factor by each PAF analysis). Concerning 12‐item PAF analysis, Cronbach's alpha of the first factor was 0.912. Concerning 10‐item PAF analysis, Cronbach's alpha of the first factor was 0.809.

## 4 Discussion

In this study the translation, cultural adaptation, and preliminary validation of the Italian version of the ATS was presented; its original 12 item structure was maintained (Hinshaw and Atwood 1983). Scores ranged from 12 to 60 points, as items were scored on a 5‐point Likert scale (Cheng et al. 2014), with higher scores reflecting a greater intention to leave the current position. It is important to note that items 1, 3, 6, 8, 9, and 10 were phrased negatively and required reverse scoring in data analysis. The scale measured turnover intention specifically and did not assess the intention to leave the nursing profession.

### 4.1 Content and Structural Validity

Concerning content validity, the S‐CVI of the Italian version of the ATS was 0.68, indicating a lower level of overall expert agreement compared with a Turkish validation study, in which the S‐CVI was calculated using the Davis technique with a panel of 10 experts and reached 0.95 (Yeşilyurt Sevim et al. 2025). In our study, only items 2 and 12 showed full and statistically significant agreement among experts. Also, items 1, 5, 6, and 11 showed high levels of agreement, although these values did not reach statistical significance. Particular concern was raised regarding items 7 and 9, as most evaluators deemed them not relevant to the measured construct, resulting in very low I‐CVI values (0.17). When items 7 and 9 were excluded from the S‐CVI calculation, the index increased to 0.78. Such findings suggest that these items need further consideration to achieve appropriate content validity and cultural consistency of the 12‐item Italian version of the ATS presented here. However, although previous literature recommended at least five experts to compose the panel (Gélinas et al. 2008), the number of six evaluators enrolled for the present investigation could be relatively small. Therefore, findings on content validity indexes should be interpreted with caution. Actually, with such a small panel, even a single disagreement can substantially affect both I‐CVI values and statistical significance, making the content validity estimates particularly sensitive to individual ratings.

The factor analysis of the 12‐item ATS scale identified three main factors. The first represented a general attitude towards remaining in or potentially leaving the current job position. It appeared to primarily reflect internal turnover within the organization, with items exploring the willingness to stay or consider other job opportunities within the same organization. Eight out of 12 items loaded positively, except for item 4 (positive loading lower than 0.30), item 7 (negative loading higher than 0.30), and items 9 and 11 (negative loadings lower than 0.30). The second factor seemed more closely related to external turnover, expressing a greater inclination to leave the organization for other opportunities. Item 11, which refers to the expression of serious doubts to remain in the current organization, is completely represented by such factor and the other two items with a positive loading higher than 0.30 (4 and 5) reflected considerations of new job prospects. Finally, the third factor, which explained a smaller portion of the variance, was strongly associated only with item 9, that was characterized by uncertainty and indecision regarding the duration of the current role.

The factor analysis of the 10‐item version identified two main factors. The removal of two items led to a small increase in explained variance and the extracted factors seemed very similar to the first two factors of the 12‐item version factor analysis. The first factor, from which items 4 and 11 remained excluded, seemed again to reflect internal turnover within the current organization. In the second extracted factor, items 5 and 11 showed loadings higher than 0.30 and seemed to express, on the contrary, the intention to leave the current organization (e.g., external turnover). It is worth noting that item 4 did not load in any factor, although loadings were positive, but slightly lower than 0.30, on both factors.

Future studies should perform a confirmatory factor analysis with an independent sample to confirm the factor structure reported here.

### 4.2 Reliability as Internal Consistency

The 12‐item ATS scale showed a very good internal consistency. Excluding items 7 and 9 further improved the scale's reliability. Internal consistency of the first extracted factor by each one of the two PAF analyses performed was substantially similar. While excluding certain items could enhance reliability, the decision to still take into account all items is driven by the aim of achieving a comprehensive, although initial, validation of the scale in a new cultural setting.

### 4.3 Comparison With Other Validation Studies

In the original study by Hinshaw and Atwood (1983), factor analysis identified two factors accounting for 54.9% of the variance. Sul and Lucas (2020) found two factors explaining 58.9% of the variance in the 12‐item version, while the 10‐item version revealed a single factor explaining 58.2% of the variance. Our study identified three factors in the 12‐item version explaining 57.82% of the variance, and two factors in the 10‐item version explaining 59.16%; both solutions seemed to prevalently reflect a distinction between internal and external anticipated turnover. This suggests a different factor structure compared to previous validations, which explains, at the same time, an equivalent proportion of variance. The Hinshaw and Atwood study involved 1.525 professionals from 15 rural hospitals in Arizona, while de Sul and Lucas studied 259 nurses in Lisbon, making this latter sample more comparable to ours. The Portuguese validation included confirmatory factor analysis, which improved model fit after some adjustments. Comparing validations across different languages and cultural contexts, over a period of 40 years, requires careful consideration of cultural and linguistic influences. Cronbach's alpha in Hinshaw and Atwood (1983) was 0.84. Sul and Lucas (2020) reported 0.87 for the 12‐item version, increasing to 0.91 after removing items 4 and 9. Barlow and Zangaro's (2010) meta‐analysis showed alphas ranging from 0.85 to 0.94, with a mean of 0.89. Our study's Cronbach's alpha coefficients were slightly lower (0.83 for 12‐items, 0.90 for 10‐items, 0.91 and 0.81 for each first extracted factor by the two PAF analysis performed), but still largely acceptable. The increase in such coefficient after excluding items 7 and 9 aligns with de Sul and Lucas's findings, highlighting the need for cautious interpretation of internal consistency due to study variations and item exclusions.

### 4.4 Implications for Nursing Practice

The study of turnover intentions can guide strategies to promote retention. The availability of a validated instrument to assess intention to leave can help nursing managers in monitoring this phenomenon and plan targeted and personalized interventions with the goal to enhance job satisfaction and reduce turnover. Moreover, it stimulates key insights for improving retention, engagement, and well‐being among nursing professionals. However, the Italian version of ATS proposed here is not ready for practical application yet, and further psychometric evaluation is needed.

### 4.5 Limitations

This study has some limitations. Beyond the need to deepen the validation process of the presented Italian version of ATS and make its application possible in practical contexts, the absence of a confirmatory factor analysis represents a further limitation, as it would have allowed confirming the observed factor structures and assessing the model fit. Criterion validity could not be assessed due to the lack of an Italian gold standard for turnover measurement. Additionally, establishing a cut‐off point for turnover intention was not feasible due to privacy and timing constraints. Temporal stability remains uncertain, as the literature does not provide guidance on the appropriate interval for repeated administration. While translation and back‐translation followed established procedures, using only certified translators or conducting the process in groups might have enhanced accuracy. The expert committee, consisting of six professionals, may not have fully captured content validity, and a larger panel could have been beneficial. Finally, a pilot test with interviews could have identified comprehension issues and informed further adjustments.

## 5 Conclusions

This study provides a preliminary validation of the ATS for the Italian context, offering first data on its content validity, structural validity, and reliability as internal consistency. However, it is not currently ready for practical application yet, although content validity and internal consistency results make the 10‐item version closer to its use by the nursing management. Future studies should further deepen its psychometric evaluation and perform a confirmatory factor analysis with an independent sample to confirm the factor structures reported here. They should also focus on the test–retest reliability of the ATS and establish a cut‐off point by relating scores with actual turnover. Further cultural adaptation and validation in the Italian context is necessary to enhance ATS psychometric validation, along with additional studies to validate the 10‐item scale proposed here. Additionally, exploring staff retention strategies, including professional development, well‐being programs, and psychological support, is essential for best practices.

## Author Contributions

**Sara Cignola:** conceptualization, methodology, validation, investigation, writing – original draft, writing – review and editing. **Stefano Bembich:** conceptualization, methodology, validation, formal analysis, writing – review and editing. **Gianfranco Sanson:** conceptualization, methodology, validation, visualization, writing – review and editing.

## Funding

The authors have nothing to report.

## Disclosure

The data reported in this manuscript have not been published previously, nor are they under consideration for publication elsewhere.

The authors have checked to make sure that their submission conforms as applicable to the Journal's statistical guidelines. The statistical analyses were reviewed prior to submission by an expert in psychometric analysis who is a member of the research team (Stefano Bembich). The authors affirm that the statistical methods used have been appropriately applied within the study design and context, and that the statistical findings have been correctly implemented and interpreted. They also accept responsibility for ensuring that the choice of statistical approach is appropriate and that all analyses have been conducted and interpreted correctly, in accordance with the Journal's submission requirements.

Some of the references cited are older than 5 years because they refer to specific, foundational studies for which no more recent equivalents were found. Moreover, several of them are key methodological sources required for the validation of the instrument.

The study was reported in accordance with the Strengthening the Reporting of Observational Studies in Epidemiology (STROBE) checklist.

## Ethics Statement

The Board of Directors of the selected hospital authorized the study. Given its observational nature, the type of data collected and the absence of any intervention involving partecipants, formal ethical approval was not required.

## Conflicts of Interest

The authors declare no conflicts of interest.

## Supporting information


**Table S1:** Demographic and professional characteristics of the study population.

## Data Availability

The data that support the findings of this study are available from the corresponding author upon reasonable request.
