# Supplementary material for: Evaluation of Nurses' Intent to Leave: Italian Translation, Cultural Adaptation, and Initial Psychometric Assessment of the Anticipated Turnover Scale
Source: Nurs Open. 2026 Oct 1;13(10):e70848. doi: 10.1002/nop2.70848 (PMC13628290; doi:10.1002/nop2.70848)
Supplement: Supplementary file 1 [file NOP2-13-e70848-s001.docx]

**Supplementary table. Demographic and professional characteristics of the study population**

| **Variable** | **n** | **%** |
| --- | --- | --- |
| Gender |  |  |
| Male | 39 | 23.2 |
| Female | 128 | 76.2 |
| Prefer not to answer | 1 | 0.6 |
| Nursing education level |  |  |
| Bachelor's degree (or equivalent) | 121 | 72% |
| First-level master's degree | 40 | 23.8 |
| Master's degree | 6 | 3.6 |
| Second-level master's degree | 1 | 0.6 |
| PhD | 0 | 0 |
| University degrees in other disciplines |  |  |
| Yes | 14 | 8.3 |
| No | 154 | 91.7 |
| Clinical area of affiliation |  |  |
| Critical care | 66 | 39.3 |
| Medical Area/Specialist Medicine | 52 | 31 |
| Surgical Area/Specialist Surgery | 23 | 13.7 |
| Outpatient Clinics and/or Services | 17 | 10.1 |
| Operating Rooms | 10 | 6 |
| Night shift |  |  |
| Yes | 112 | 66.7 |
| No | 56 | 33.3 |
| 12-hour shifts |  |  |
| Yes | 73 | 43.5 |
| No | 95 | 56.5 |
| Marital status |  |  |
| Living with a partner | 52 | 31 |
| Married | 54 | 32.1 |
| Single | 45 | 26.8 |
| Other | 8 | 4.8 |
| Prefer not to answer | 6 | 3.6 |
| Widowed | 3 | 1.8 |
| Number of children |  |  |
| None | 93 | 55.4 |
| One | 28 | 16.7 |
| Two | 34 | 20.2 |
| Three | 11 | 6.5 |
| Four | 2 | 1.2 |
